# Comparative study on the kinetic model and effect of sludge protein dissolution under the synergistic action of acid-thermal and alkaline-thermal

**DOI:** 10.1371/journal.pone.0339182

**Published:** 2026-02-02

**Authors:** Zheng Li

**Affiliations:** Zhengzhou University Multi-Functional Design and Research academy Co., LTD., Zhengzhou, China; North-Caucasus Federal University, RUSSIAN FEDERATION

Sludge protein has been widely used in daily life. In this paper, based on the acid-base mechanism and kinetic model theory, kinetic models for the temperature, time, sludge concentration and acid-base concentration of the alkaline heating method and the acidic heating method were established. The kinetic model parameters were obtained using nonlinear optimization technology. The acid-base hydrolysis process and the influence laws of various factors were further analyzed based on the data model and parameters. The results show that the residual sum of squares (SSE) of the optimized parameter data of the data model obtained under acidic heating conditions is 6.37 × 10^7^, and the square sum of the correlation coefficient (R^2^) is 0.848; the residual sum of squares (SSE) of the optimized parameter data of the data model obtained under alkaline heating conditions is 2.828 × 10^7^, and the square sum of the correlation coefficient (R^2^) is 0.892. The data models obtained under the above two conditions more accurately describe the protein extraction process, and the data effect is good. Through the comparison of data parameters in the above kinetic research process, it is applied to the extraction of sludge protein. In the generation process of peptides and amino acids, the influence of sulfuric acid is greater than that of sodium hydroxide. Perhaps the acidic heating method is more conducive to promoting protein hydrolysis.

## Introduction

As the living standards of residents continue to improve, the burden on municipal sewage treatment plants becomes increasingly heavy, and the secondary treatment of sludge has become an urgent matter [1]. Currently, the application of sludge is mainly used in urban greening. However, since sludge contains high concentrations of proteins [2], some experts have proposed extracting the proteins from the sludge and applying them to foaming agents, fertilizers, animal feed, etc., to achieve the recycling of high-value resources [3–6]. There are various methods for extracting sludge proteins, such as hot hydrolysis method, ultrasonic method, chemical method, and biological method [7–11]. Currently, the chemical method is the most widely used method, and there are acid-thermal and alkali-thermal in chemistry [12].

The composition of activated sludge is complex and mainly consists of bacterial flocs [13]. The bacterial flocs are mainly Gram-negative bacteria [14,15]. Adding acid or alkali to the sludge will accelerate the hydrolysis of proteins or lipoproteins in the cell wall, causing the cell wall to rupture and the organic matter to dissolve [16]. Therefore, in the process of extracting protein from sludge, acid/alkali is often used as a catalyst. During the acid/alkali hydrolysis process of the sludge, in addition to destroying the cell wall and dissolving proteins, it will also break down proteins into small molecule peptides, and then generate amino acids [17,18]. Therefore, the protein extraction process in sludge includes the process of dissolving the cell wall to dissolve proteins and hydrolyzing proteins to generate peptides and amino acids [19].

There have been many reports in the literature on methods of extracting protein from sludge [20,21]. Shier used thermal extraction to extract protein from sludge [22]. The results showed that the optimal conditions for extracting protein in the experiment were T = 150–155°C, t = 20 min. Under the experimental conditions, macromolecular proteins can be obtained; Cui Jing [23] and others have conducted an in-depth study of the effects of pH, temperature, water content and time on protein extraction through experiments with two different sources of sludge. The results show that the protein recovery rate is as high as 61.34% in an alkaline heat environment. The optimal hydrolysis conditions are: pH = 13, T = 140°C, W = 91%, t = 3 h. Liu Yulei [24] studied the recovery of protein in the sludge of municipal sewage treatment plants, and the results showed that temperature has a significant effect on the recovery of protein by strong acid heating, followed by time and pH, and the optimal hydrolysis conditions are as follows: T = 120°C, pH = 2, time t = 4 h, water content W = 87%, under these conditions the protein recovery rate is 48.12%. D.J.Leeand others studied the cracking of sludge cells by low-intensity ultrasound, and explored the promotion of anaerobic digestion at medium temperature by this process [25]. The results show that when the ultrasonic frequency is 20KHz and the sound intensity is 0.33W/ml, the SCOD/TCOD in the sludge only rises from 0.06—0.08 to 0.1 when the sludge is cracked for 20 minutes. Hwang conducted hydrolysis experiments on sludge through the combined use of ultrasound and alkali, and the results showed that the maximum protein content of sludge was 3177.5 mg/L under the experimental conditions [26]. Among these methods for extracting sludge protein, acid-thermal and alkali-thermal have become the mainstream technology because of their good lysis effect and high quality of extracted protein. However, due to the complicated process of sludge lysis, there are few reports on the kinetic model of sludge protein dissolution [7,27]. In particular, the comparison of the kinetic model of sludge protein dissolution is used to compare and analyze the protein dissolution effect of acid-thermal and alkaline heat method Rare.

In this paper, the acid/base concentration, time, temperature and water content are used to study the factors, and the corresponding mathematical model is established through the change of protein concentration. The data in the experiment process is adopted by Levenberg-Marquardt in the opt software. The global optimization method performs nonlinear optimization to calculate the optimized model parameters, analyze the kinetic model to understand the process of sludge protein extraction and provide basic mathematical theory basis. Subsequently, in the process of extracting the protein from the sludge using the optimized acid-thermal and alkali-thermal models, it significantly increased the extraction rate of sludge protein compared to the current methods used in wastewater treatment plants. This might be attributed to the fact that the optimized acid-thermal enables more microbial cell structures to be lysed, releasing more organic substances.

## Materials and methods

### Source of sludge

The sludge used in the experiment was concentrated sludge from a municipal sewage treatment plant in Zhengzhou, which was a mixed sludge including sludge in the primary sedimentation tank and sludge in the secondary sedimentation tank, with a moisture content of about 94.5%, and then added 1‰ of PAM. Centrifugal concentration. The characteristics of the test sludge and its supernatant are shown in Table 1. The remaining three sludges come from the sewage treatment plants in Kaifeng City, Zhouzou City and Xinxiang City.

**Table 1:** Properties of the original sludge and the original sludge supernatant.

Project	Parameter	Value
Sludge	MLSS (g/L)	60.255 ± 0.64
	MLVSS (g/L)	39.125 ± 1.45
	TCOD (mg/L)	56002.7 ± 3478.4
	TKN (mg/L)	2508 ± 771
Supernatant	pH	6.7 ± 0.5
	COD (mg/L)	476.9 ± 69
	Protein (mg/100g)	281.63 ± 43.5

The reaction vessel and parameter settings for the sludge treatment through hydrolysis reaction were consistent with those set by Jianlei Gao [28]. The instruments for other test indicators are shown in Table 2.

**Table 2:** List of commonly used equipment.

Equipment	Model	Manufacturer
BCA kit	P1511	APPLYGEN Gene Technology Co., Ltd.
pH meter	PHSJ-4A	Shanghai Precision Scientific Instrument Co., Ltd.
Centrifuge	TDZ5-WS	Hunan Xiangyi Laboratory Instrument Development Co., Ltd.
Solvent filter	1L standard type	Tianjin Jinteng Instrument Co., Ltd.
Diaphragm pump	GM-1.0A	Tianjin Jinteng Instrument Co., Ltd.
Spectrophotometer	Gury60	Shanghai CSOIF Co., Ltd
Freezer	BD/BC-265VMQ	Hefei Hualing Co., Ltd.

### Determination of crude protein concentration

After the reaction, the sludge hydrolysate was centrifuged at 4000–5000 rpm/min for 15 min. Then, 100 mL of the supernatant was taken for the Kjeldahl nitrogen determination test.

### Determination of soluble proteins

liquid supernatant the protein concentration of the filtrate was tested using a protein quantification kit (BCA method, Beijing Pulilai Gene Technology Co., Ltd.).

### Determination of polysaccharide concentration

Take 1 mL of the supernatant from the hydrolysis process, add 1.0 mL of distilled water to it, then add 1.0 mL of 6% phenol, quickly add 5.0 mL of concentrated sulfuric acid, shake it on a vortex mixer, mix thoroughly, let it stand for 30 minutes, measure the absorbance at 490 nm, and input the measured absorbance into the standard curve to obtain the concentration of the polysaccharide.

### Protein secondary structure

The isolated proteins were vacuum freeze-dried at −80°C for 48 hours. After grinding, Fourier transform infrared spectroscopy was used to scan the samples in the wavelength range of 4000–400 cm-1. The data related to protein secondary structure were obtained through analysis using PeakFit software.

### SCOD concentration test

The upper clear liquid was tested using the COD kit(Therom Fisher, CODS10).

### Zeta potential

The hydrolysate was analyzed using a Zeta potential and nanoparticle size analyzer.

### Protein precipitation

In an ice bath, add solid ammonium sulfate to the protein solution and continue stirring. Adjust the solution to a saturated concentration of 80% ammonium sulfate. After complete dissolution of ammonium sulfate, place the solution in a 4°C refrigerator and let it stand for 12 hours to achieve precipitation and layering; then, centrifuge at 8000 revolutions per minute and 4°C for 10 minutes.


$$Protein\;precipitation\;rate(\%)=\frac{C_{\text{1}}-C_{\text{2}}}{C_{\text{1}}}\times \text{1}\text{0}\text{0}\%$$ (1)

In the formula: C_1_: The protein content in the solution before precipitation (mg/L); C_2_: Protein content in the solution after precipitation (mg/L).

### Determination of pH

The pH adjustment is measured with a pH meter (model: PHSJ-4A, Shanghai Precision Scientific Instrument Co., Ltd.).

## Results and discussion

### Overview and establishment of dynamic model

The extraction process of sludge protein mainly involves lysing the sludge cells to release proteins, polysaccharides and other organic substances, and then further decomposing and extracting peptides and amino acids. The analysis of the above protein extraction process, it can be seen that the process not only includes the process of breaking the wall to extract the protein, but also the process of proteolysis to produce small peptides and amino acids. This process is affected by acid/alkali concentration, temperature, time and water content during the hydrolysis process.

At present, the reaction rate equation that is mainly reflected in the hydrolysis process of polymers such as cellulose can be simplified into a first-order reaction model [29–31]. Therefore, in this study, the process of dissolving the cell wall to extract protein can be simplified as a first-order reaction model of bacterial concentration, and the hydrolysis of protein can also be a first-order reaction process of protein concentration. Since water is one of the necessary factors in the hydrolysis process, the water content of the hydrolyzed sludge in this study is in the range of 90%−96%, and the water content is large. The water concentration can be visualized in the reaction kinetics. Is a constant. Since the source of sludge used in this experiment is municipal sludge, the source of sludge is stable, so the sludge concentration can be used to represent the biological cell concentration.

The reaction of the process of extracting protein by hydrolysis can be expressed by the following formula:


$$-\frac{dC_{S}}{dt}=k_{f}C_{s}$$ (2)

In the formula $C_{S}$ --- sludge concentration (g/kg)

t------Reaction time (s)

$k_{f}$ ---Response rate constant (s^-1^)

It can be obtained by formula (1)


$$C_{s}=C_{s,0}e^{-K_{f}t}$$ (3)

$C_{S,0}$ is the initial sludge concentration (g/kg)

The rate of generation of sludge cell wall cracking and releasing protein is:


$$\frac{dP}{dt}=K_{f}k_{2}C_{s}$$ (4)

P---protein concentration in the formula (mg/L)

$K_{1}$ ---The product of the ratio coefficient between the sludge reaction quality and the protein production quality and the ratio coefficient between the standard protein separation amount and the molecular weight of the protein extracted by the reagent (mg·Kg/(g·L))

In this experiment, the hydrolysis rate of protein is expressed in direct proportion to the first power of protein concentration.


$$-\frac{dP}{dt}=K_{S}P$$ (5)

$K_{S}$ ---Protein hydrolysis rate ($S^{-1}$);

From (3) and (4), the protein production rate equation of sludge in the process of acid-base interaction can be obtained:


$$\frac{dP}{dt}=K_{f}k_{2}C_{s}-K_{S}P$$ (6)

Sort out the non-homogeneous ordinary differential equations:


$$\frac{dP}{dt}+K_{S}P=K_{f}k_{2}C_{s,0}e^{-K_{f}t}$$ (7)

The general solution of this non-homogeneous ordinary differential equation is as follows:


$$P=\frac{K_{f}k_{1}C_{s,0}}{K_{s}-K_{f}}e^{-K_{f}t}$$ (8)

Formula (7) is the relationship between the influence of t (time), c (acid-base concentration), T (temperature), and C (sludge concentration) on the protein concentration in the process of hydrolyzing sludge by acid-thermal/alkaline-heat method.

For the determination of most chemical reaction rate constants, it is generally believed that it conforms to the Arrhenius formula:


$$K=Ae^{-E_{a}/RT}$$ (9)

A---Pre-reference factor

E_a_----Activation energy, the rate constant of reaction degree K is related to activation energy

Based on the Arrhenius formula, combined with the collision theory, the transition state theory obtained is to express the reaction rate constant as the following formula.


$$K=\frac{RT}{N_{0}h}e^{\Delta S_{a}/R}e^{-E_{a}/RT}$$ (10)

Honggang believed that the catalytic effect of the catalyst was achieved by reducing the activation energy Ea and changing the pre-exponential factor A, where A depends on the reaction entropy change Sa [32]. Therefore, in order to facilitate data processing, the empirical formula can be simplified as the following formula:


$$K_{f}=k_{f0}e^{-E_{1}/RT}e^{f_{1}C}$$ (11)

$$K_{s}=k_{s0}e^{-E_{2}/RT}e^{f_{2}C}$$ (12)

k_f,0_、k_s,0_-----Factor before rate constant (S^-1^)

E_1_、E_2_-----Activation energy(J/mol)

f_1_、f_2_----The rate constant’s acid-base index influence coefficient (Kg/g)

C-----Acid or alkali concentration. In this experiment, the acid is sulfuric acid and the alkali is sodium hydroxide (g/Kg)

### Model parameter optimization

1) **Optimization of acid heat method data model**

**Fig 1:**
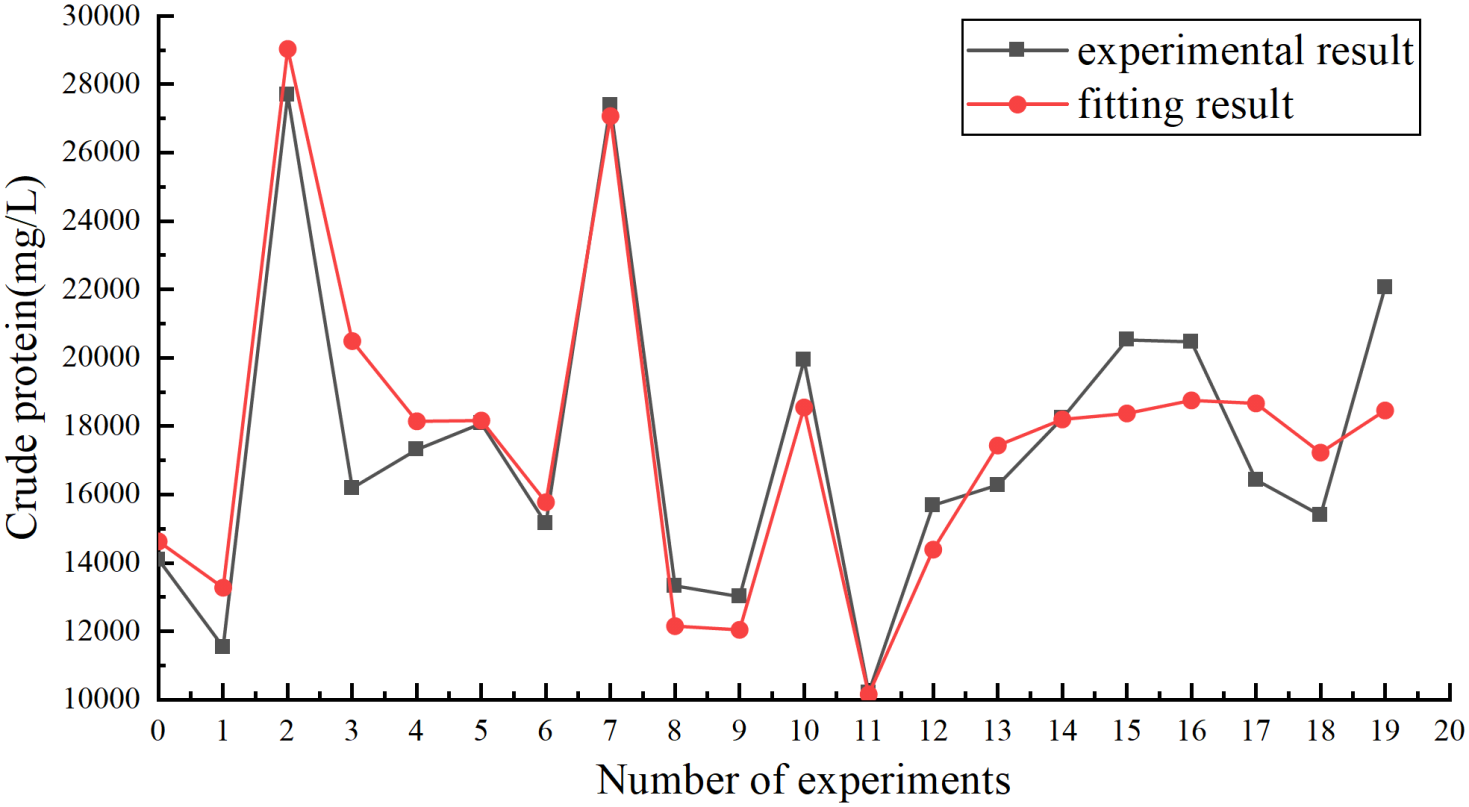
Nonlinear fitting results of acid-thermal data model.

After 20 sets of experimental data in this study, opt software was used to optimize 7 data parameters using nonlinear optimization techniques. The optimization results of the 7 parameters K_f,0_, K_s,0_, K_1_, E_1_, E_2_, f_1_, f_2_ are: 60.59 S^-1^, 14.37 S^-1^, 220.61 mg/g, 0.288 J/mol, 1.533 J/mol, 0.463 Kg/g, 0.452 Kg/g. The effect diagram of the data model obtained with opt is shown in Fig 1.

The residual sum of squares (SSE) of the optimized parameter data of the obtained data model is 6.37 × 10^7^, and the correlation coefficient sum of squares (R^2^) is 0.848. The results show that this data model is more accurate in describing the process of extracting protein by acid-thermal, and the data effect is excellent From the parameter values in the kinetic data model, it can be seen that E_1_ and E_2_ are 1.488 J/mol and 1.53 J/mol, respectively, which are greater than 0, indicating that the process is an endothermic reaction, and increasing the temperature is beneficial to the progress of the sludge reaction process. Release more protein and promote protein hydrolysis. It can be seen from f_1_ and f_2_ that the values are 0.463 Kg/g and 0.452 Kg/g, both of which are positive, indicating that the addition of sulfuric acid can promote the cracking of the cell wall, so that more protein can be dissolved. The experimental process represented by the above values is consistent with the performance of the experimental phenomenon. The optimization process of the obtained kinetic data model is reasonable. The applicable range of the parameters of the data model: temperature 90–140°C, sludge concentration 30-95g/kg, concentrated sulfuric acid concentration 9- 20g/kg, time 0-18000s. The experimental data is shown in Table 3.

**Table 3:** The experimental protein concentration under various conditions.

pH (g/kg)	Time (s)	Temperature (°C)	C (g/kg)	Protein Concentration(mg/L)
16.56	18000	140	40.97	14724
12.88	10800	130	41.98	13380
12.88	14400	140	88.99	29174
12.88	18000	120	66.11	20574
9.2	10800	140	61.48	18211
27.6	10800	120	66.58	18123.23
9.2	10800	120	67.9	15759.72
16.56	14400	130	90.88	27235.69
5.52	10800	120	64.88	12161.12
16.56	10800	100	64.11	12112.62
16.56	14400	130	65.98	18663.38
9.2	14400	120	45.79	10189
16.56	10800	110	66.92	14466.46
16.56	10800	120	60.9	17512.62
16.56	10800	130	60.34	18189.54
16.56	18000	130	67.89	18463.38
16.56	14400	130	67.88	18792.62
16.56	10800	120	61.48	18687.44
12.88	10800	120	62.8	17275.51
9.2	18000	130	88.89	18589

Footnotes: The experiment of extracting sludge protein was conducted by varying four parameters: pH, reaction time, temperature and sludge content. The relationship between the relevant parameters and the protein extraction rate was obtained.

2) **Optimization of the Alkaline Heating Method Data Model**

**Fig 2:**
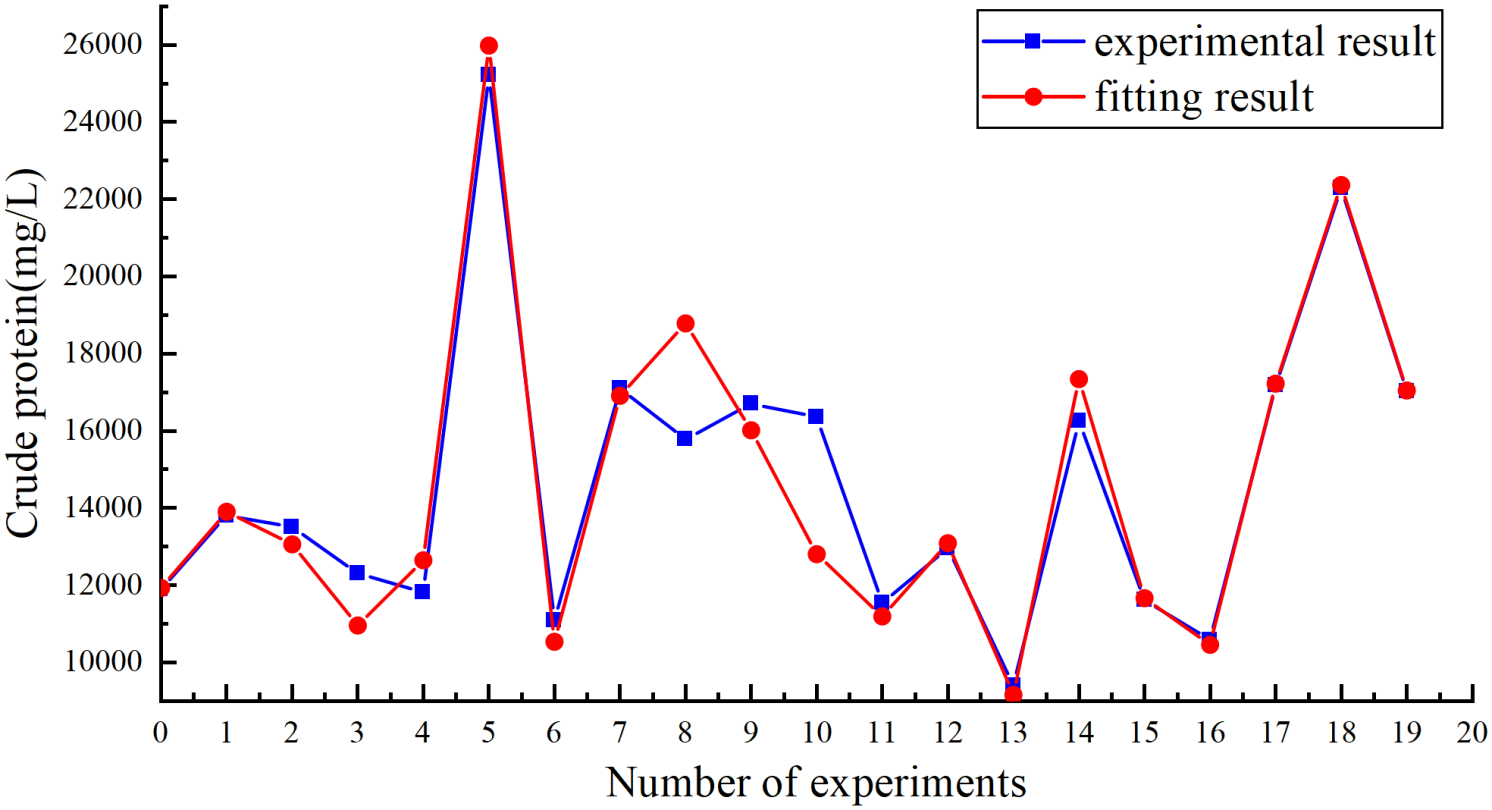
Non-linear fitting diagram of the alkaline heating method data model.

After 20 sets of experimental data in this study, opt software was used to optimize 7 data parameters using nonlinear optimization techniques. The optimization results of the seven parameters K_f,0_, K_s,0_, K_1_, E_1_, E_2_, f_1_, f_2_ are: 39.22 S^-1^, 0.047 S^-1^,5236.7 mg/g, 0.453J/mol, 0.004J/mol, 0.384 Kg/g, 0.111 Kg/g. The effect diagram of the data model obtained with opt is shown in Fig 2.

The residual sum of squares (SSE) of the optimized parameter data of the obtained data model is 2.828 × 10^7^, and the correlation coefficient sum of squares (R^2^) is 0.892. The results show that this data model is more accurate in describing the process of extracting protein by acid-thermal. Excellent. According to the parameter values in the kinetic data model, E_1_ and E_2_ are both greater than 0, indicating that the endothermic reaction occurs during the process. At the same time, increasing the temperature is beneficial to the sludge reaction process, releasing more protein, and promoting Hydrolysis of protein. From f_1_ and f_2_ respectively, it can be known that 0.384 Kg/g and 0.111 Kg/g, and the value is greater than 0, indicating that the addition of sodium hydroxide can promote the cracking of the cell wall to a certain extent, promote the progress of the reaction, and indicate that sodium hydroxide It can also promote the hydrolysis of proteins. The experimental process represented by the above values is basically consistent with the performance of the experimental phenomenon. The optimization process of the obtained kinetic data model is reasonable. The applicable range of the parameters of the data model: temperature 90–140°C, sludge concentration 30-95g/kg, sodium hydroxide concentration 0-4g/kg, time 0-18000s. The experimental data is shown in Table 4.

**Table 4:** The experimental protein concentration under various conditions.

pH (g/kg)	Time (s)	Temperature (°C)	C (g/kg)	Protein Concentration(mg/L)
1	10800	120	68.5	11987.09
2	10800	110	91.34	13999.92
2	14400	120	68.51	13077.23
2	18000	130	45.67	11001.08
1.6	10800	120	66.4	12722.53
3	14400	130	92.55	26098.77
3	10800	120	44.88	10580.31
3	10800	120	69.9	16924.63
3	10800	120	64.4	18888.14
3	10800	140	68.9	16197.61
3	7200	120	66.9	12865.68
3	18000	110	68.21	11183.38
2	10800	120	65.9	13199.72
3	10800	100	66.6	9209.544
3	10800	120	66.5	17421.47
3	10800	110	68.9	11756.91
4	14400	110	47.77	10503.38
4	10800	120	65.8	17309.19
3	14400	120	91.22	22461.47
3	14400	120	68.8	17014.46

Footnotes: The experiment of extracting sludge protein was conducted by varying four parameters: pH, reaction time, temperature and sludge content. The relationship between the relevant parameters and the protein extraction rate was obtained.

### Comparison of the kinetics of the process of extracting protein by acid heat method and alkali heat

Through the above kinetic research process, it is possible to compare the rate of acid-heating and alkaline-heating hydrolysis processes through data parameters to clarify the degree of influence of these two processes.

In the process of sludge cell cracking, when concentrated sulfuric acid is used as a catalyst in a high temperature environment, the concentration index of concentrated sulfuric acid in the rate constant has an influence coefficient of 0.463 Kg/g, which is relative to the alkali concentration index when sodium hydroxide is used as a catalyst. The influence coefficient is large and positive, indicating that concentrated sulfuric acid has a great effect on the sludge cell cracking process. At the same time, adding concentrated sulfuric acid can promote the sludge cell cracking and dissolve more protein. This shows that the hydrolysis reaction when concentrated sulfuric acid is the catalyst is easier to crack the cell wall of the sludge and release more protein, which is consistent with the research phenomenon. Since the reaction rate constant of concentrated sulfuric acid obtained in this study is greater than that of sodium hydroxide when the experimental temperature is 90–140°C, the activation energy is positive, and increasing the hydrolysis temperature can promote the hydrolysis process. The exponential factor of the hydrolysis reaction under the sodium hydroxide condition is smaller than that under the concentrated sulfuric acid condition, and the hydrolysis catalytic rate under acidic conditions is greater than that under alkaline conditions. The influence coefficients of the rate constant of concentrated sulfuric acid and sodium hydroxide in the hydrolysis of protein are 0.452 kg/g and 0.111 kg/g, respectively. Therefore, the influence of concentrated sulfuric acid in the process of hydrolyzing protein into peptides and amino acids is greater than that of sodium hydroxide. Under this experimental condition, the acid-thermal is easier to promote the dissolution of sludge protein.

**Fig 3:**
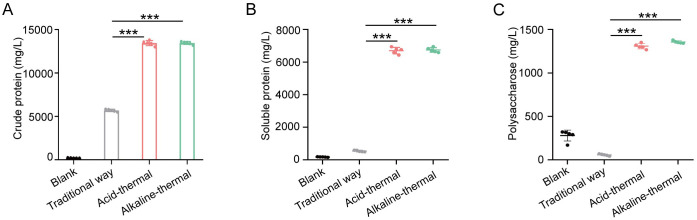
The effects of different methods on the crude protein(A), soluble protein(B) and polysaccharides(C) in the supernatant after sludge treatment.

The previous section discussed the model optimization for the acid-thermal and the alkali-thermal. In order to verify the application value of the optimized model in helping wastewater treatment plants extract more sludge protein. Experiments on extracting protein from sludge from the same source were conducted using the traditional method of the sewage treatment plant, as well as the optimized acid-thermal and alkali-thermal. As shown in Fig 3A, the crude protein content extracted by the optimized acid-thermal and alkali-thermal is much higher than that extracted by the traditional methods. The crude protein content of these methods is approximately three times that of the traditional methods. Furthermore, for the crude protein, a further extraction was carried out. For the soluble protein, the extraction yields by the acid-thermal and the alkali-thermal were approximately 7 times that of the traditional methods (Fig 3B). By analyzing the extraction amounts of crude protein and soluble protein, it can be clearly observed that the optimized acid-thermal and alkali-thermal are more conducive to the extraction of soluble protein. Furthermore, the determination of the polysaccharide content in the supernatant is also a key indicator for extracting sludge proteins. As shown in Fig 3C, it can be observed that the content of polysaccharides extracted by the optimized acid-thermal and alkali-thermal is much higher than that of the traditional methods, and the trend is consistent with that of soluble proteins.

Furthermore, the extracted supernatant was subjected to three-dimensional fluorescence intensity observation (Table 5). The optimized acid-thermal and heating method showed significantly enhanced fluorescence intensity in all regions compared to the traditional methods, with an overall increase of 2–3 times. Among them, the proteins in the second region have simple directions. The fluorescence intensity after optimization has increased by 3.34 to 3.45 times. The proof is that the optimized acid-thermal and alkali-thermal facilitate the release of protein from the sludge.

**Table 5:** The influence of different methods on the relative intensity of three-dimensional fluorescence in the supernatant after sludge treatment.

100 mL liquid supernatant	I	II	III	IV	V
Traditional method	1	1	1	1	1
acid-thermal	1.78	3.45	3.58	2.73	3.41
alkali-thermal	1.71	3.34	3.47	2.62	3.33

Footnotes: After the supernatants extracted by different methods were subjected to freeze-drying treatment, Fourier transform infrared spectroscopy was used to scan the samples within the wavelength range of 4000–400 cm-1. The data related to the secondary structure of proteins were obtained through analysis using PeakFit software.

**Fig 4:**
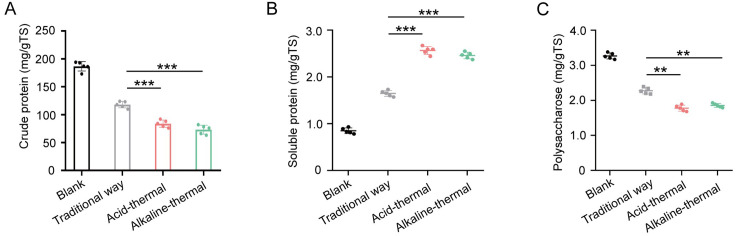
The effects of different methods on the crude protein(A), soluble protein(B) and polysaccharides(C) in the sludge after sludge treatment.

By testing the supernatant, it has been confirmed that the optimized acid-thermal and alkali-thermal can extract more sludge protein. To investigate the impact of the optimized method on the remaining sludge after extraction. The remaining sludge was tested, and it was found that the sludge treated by the optimized method had a much lower crude protein content than that treated by the traditional method (Fig 4A). Further analysis of the soluble proteins in the residual sludge revealed that the contents of the optimized acid-thermal and alkali-thermal were higher than those of the traditional treatment and untreated sludge (Fig 4B). This might be due to the destruction of the microbial cell walls by sulfuric acid and sodium hydroxide, resulting in an increase in soluble proteins. As for the polysaccharides in the residual sludge, the content of the optimized method was lower than that of the traditional method and the untreated sludge, which might be because more polysaccharides entered the supernatant.

### Comparison of acid-thermal and alkali-thermal

**Fig 5:**
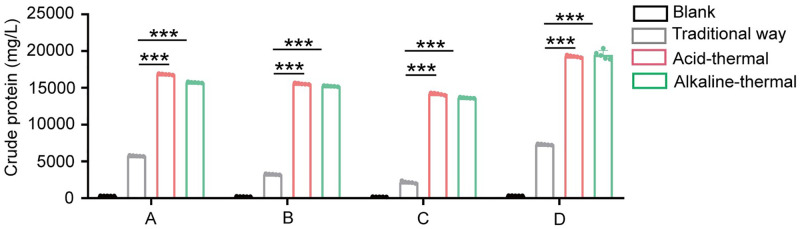
The impact of different treatment methods on the protein content of sludge from different wastewater treatment plants.

As can be observed in the previous text, the optimized acid-thermal and alkali-thermal have excellent application value in the actual extraction of sludge protein. However, there was no significant difference between the acid-thermal and the alkali-thermal in extracting sludge protein. Therefore, in order to further observe the differences between the two methods and their general application value. Collect the sludge from four wastewater treatment plants in different regions, and extract the protein from the sludge using the optimized acid-thermal and alkali-thermal. It can be observed (Fig 5) that the crude protein content of sludge varies in different regions, which might be related to the local industrial production methods. Among the sludges from the first three factories, the crude protein content extracted by the optimized acid-thermal was slightly higher than that by the hot-alkali method. However, in the sludge from Factory D, the extraction amount by the alkali-thermal was higher.

**Fig 6:**
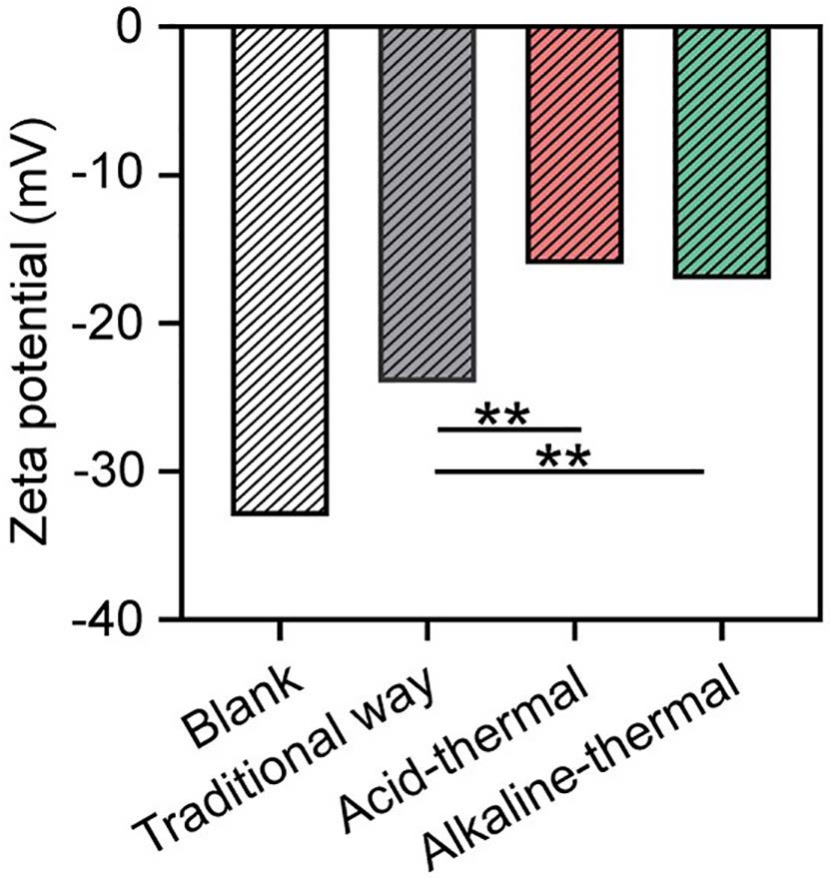
Different methods’ impact on the electrical potential of the sludge supernatant.

Zeta potential is a key indicator for evaluating the quality of sludge dewatering performance. The higher the absolute value of the residual sludge’s electric potential, the stronger the electrostatic repulsive force it exhibits, and the worse its dewatering property will be. The potential of the residual sludge from four different regions was measured. It was observed that the optimized acid-thermal method resulted in the lowest absolute potential value, while the untreated sludge had the highest absolute potential value(Fig 6).

**Fig 7:**
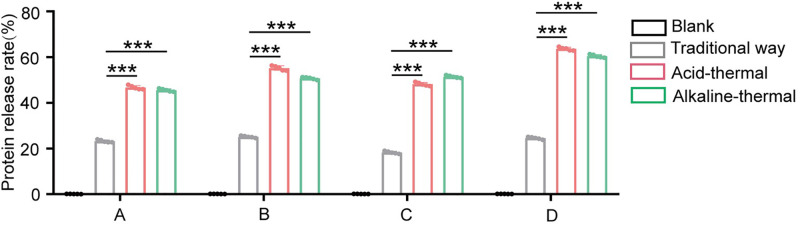
The influence of different treatment methods on the protein extraction rate of sludge from wastewater treatment plants in different regions.

Based on the different extraction yields of crude protein from sludge in four different regions obtained by the optimized acid-thermal and alkali-thermal, the soluble protein was subsequently extracted from each of them. The protein extraction rates of sludge in the four regions were then calculated using different extraction methods. As shown in Fig 7, the protein extraction rate of sludge varies among different regions using the same extraction method, which might be related to the local industrial types. However, for sludge from the same area, the optimized acid-thermal has a higher protein extraction rate than the alkaline-heat method, and is far superior to the traditional methods. This indicates that the optimized acid-thermal method has broad applicability.

**Fig 8:**
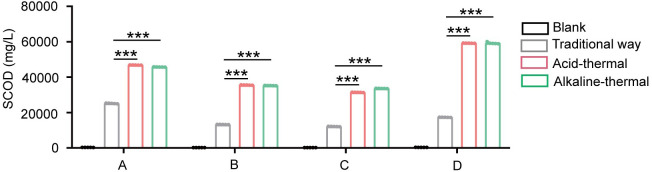
The effects of different treatment methods on the SCOD content in the supernatant of sludge from wastewater treatment plants in different regions.

A large amount of literature has demonstrated that the acid-thermal and the alkali-thermal can obtain sludge protein by destroying the cell walls of microorganisms. The concentration of SCOD in the supernatant is closely related to the degree of microbial cell destruction in the sludge. As can be seen from Fig 8, the COD content of the supernatant obtained through the optimized acid-heat treatment and alkali-heat treatment is much higher than that of the traditional methods and untreated supernatants. This is consistent with the theory. Sulfuric acid and sodium hydroxide can accelerate the destruction of the sludge flocs and microbial cell structures, allowing more organic matter to be released from the cells. Moreover, the SCOD concentration treated by the optimized acid-thermal is 3 times higher than that treated by the alkali-thermal. Therefore, whether from the perspective of model fitting or in actual protein extraction, the optimized acid-thermal is more widely applicable than the alkali-thermal.

## Conclusions

Through this experimental study, combining the structure and characteristics of the sludge, using the theory of catalyst catalytic reaction and hydrolysis reaction, a kinetic model of sludge protein dissolution under the conditions of thermal acid and thermal alkali synergistic reaction was obtained. Through experiments, the protein concentration extracted from the supernatant was obtained under different test conditions, and the sludge protein dissolution kinetic model parameters were obtained through the nonlinear optimization technology in the opt software. The results of experiments and model simulations show that the kinetic model has a better effect. The sludge protein dissolution effect obtained by the hydrolysis reaction with concentrated sulfuric acid as the catalyst is better than the hydrolysis reaction with sodium hydroxide as the catalyst. When the catalyst is used, it is easy to crack the cell wall of the sludge and release more protein. At the same time, according to the results of the kinetic model, the activation energy of the hydrolysis reaction is positive, and increasing the hydrolysis temperature can promote the hydrolysis process. Secondly, the exponential factor of the hydrolysis reaction under the sodium hydroxide condition is smaller than that under the concentrated sulfuric acid condition, so the hydrolysis catalytic rate under acidic conditions is greater than that under alkaline conditions. Therefore, the acid-thermal is easier to extract sludge protein under this experimental condition. Based on the model conclusion, further treatment was carried out on sludge from different regions. From the perspectives of protein extraction rate and SCOD indicators, the optimized acid-thermal could significantly increase the protein extraction rate of the sludge, for all the detailed data of the experiment, please refer to the supporting information (S1 File). This method can also be applied to the extraction of sludge protein from wastewater treatment plants in more regions, thereby increasing economic benefits.

## Supporting information

S1 File Detailed data of acid-thermal/alkaline-thermal for extracting sludge protein experiment. (XLSX)
